# Cardiac cycle time-corrected electromechanical activation time greater than 15% is an independent risk factor for major adverse cardiovascular events in chronic heart failure outpatients

**DOI:** 10.12669/pjms.38.3.4500

**Published:** 2022

**Authors:** Jing Zhang, Wenxian Liu

**Affiliations:** 1 Jing Zhang, MM, Division of Cardiology, Coronary Care Unit, Beijing Anzhen Hospital, Capital Medical University, Beijing 100029, P.R. China; 2 Wenxian Liu, MM, Division of Cardiology, Coronary Care Unit, Beijing Anzhen Hospital, Capital Medical University, Beijing 100029, P.R. China

**Keywords:** Cardiac cycle time-corrected electromechanical activation time (EMATc), chronic heart failure (CHF), major adverse cardiovascular events (MACEs)

## Abstract

**Objectives:**

This study aimed to investigate the predictive value of cardiac cycle time-corrected electromechanical activation time (EMATc) for major adverse cardiovascular events (MACEs) in outpatients with chronic heart failure (CHF) in comparison with other clinic indexes.

**Methods:**

This prospective observational study at Beijing Anzhen Hospital from January 01, 2015 to January 01 2018 enrolled 120 CHF patients who were admitted for acute onset of CHF and followed up after discharge for 616.5 days (range: 202.75–875.25 days). Based on the different endpoints, cardiogenic death, all-cause death, and HF-related readmission, patients were assigned to the following groups: cardiogenic death and non-cardiogenic death groups, all-cause death and survival groups, and HF readmission and non-readmission groups. EMATc and other clinic indexes were measured and compared between these groups. Cox regression analysis was used to identify independent risk factors for MACEs.

**Results:**

The hazard ratio for EMATc>15% for cardiogenic death was 3.493 (P=0.046), suggesting that an EMATc>15% was an independent risk factor for cardiogenic death in CHF patients. The hazard ratios for B-type natriuretic peptide (BNP) >400 ng/L for all-cause death and CHF readmission were 3.810 (P=0.008) and 2.764 (P=0.031), respectively. Thus, BNP >400 ng/L was an independent risk factor for all-cause death and readmission for CHF. EF<40% was not found to be a significant risk factor for MACEs.

**Conclusions:**

BNP level can predict the risk for poor prognosis in CHF patients. EMATc>15% is an independent risk factor for cardiogenic death and should be considered as a supplement to serum BNP level and other clinical indexes for predicting cardiogenic death in CHF outpatients.

## INTRODUCTION

Chronic heart failure (CHF) is the end stage of a variety of cardiovascular diseases. The high CHF mortality is mainly attributed to complications of cardiovascular etiology.^1^ Despite medical advances, the all-cause mortality of CHF is 17%, and the readmission rate is 44%.^2^ Searching for predictors of poor prognosis in CHF patients has been an active area of research. Natriuretic peptides such as B-type natriuretic peptide (BNP) have been shown to have diagnostic and predictive value for CHF^3^, and left ventricular ejection fraction (EF) is the most commonly used prognostic indicator for CHF.^4^ However, for stable CHF patients, repeated monitoring of BNP and echocardiography require considerable medical resources and increases the burden on society and the patients’ families.^5^

Acoustic cardiography is a rapid bedside test that can provide parameters reflecting left ventricular function. Electromechanical activation time (EMAT) is one of those parameters and refers to the time from the beginning of LV electrical activity (the beginning of QRS wave in electrocardiograph) to the beginning of the first heart sound (mitral valve closure).^6^ EMAT is divided by the cardiac cycle (RR interval) and can be compared for different heart rates (corrected EMAT ratio, EMATc). Elevated EMATc is considered as an indicator of decompensated HF.^7^ However, whether EMATc can be predictive for major adverse cardiovascular events (MACEs) in CHF outpatient remains to be examined. In the present study, we selected patients with CHF and investigated whether the EMATc can reliably be used to predict MACEs in patients with stable CHF in comparison to the traditional clinical indexes such as BNP and EF.

## METHODS

### Patient selection

Patients admitted to Beijing Anzhen Hospital from January 01, 2015 to January 01, 2018 for acute onset of CHF were enrolled in our study. They were admitted for worsening symptoms, which were not relieved by oral treatment.

### The inclusion criteria included

EF <50%, heart function classification of III-IV (New York Heart Association [NYHA]), and willingness to complete follow-up.

### The exclusion criteria were

age <18 years, acute myocarditis, acute myocardial infarction, plans for coronary intervention or cardiac surgery, and structural heart disease.

After application of the inclusion and exclusion criteria, these patients received standard treatment. The study protocol used in this study was approved by the Ethics Committee of Beijing Anzhen Hospital, Capital Medical University (Approval No. 2019042X). All procedures performed in this study involving human participants met the ethical standards of the institutional and national research committee and the 1964 Declaration of Helsinki and its later amendments or comparable ethical standards. Written informed consent was obtained from each study participant.

### Data Collection

All participants’ baseline data, including age, gender, medications, medical history, and MACEs that occurred during the follow-up period, were collected. The clinical data of all participants included blood pressure, resting heart rate (HR), and the levels of troponin-I (TnI), BNP and creatinine. BNP, TnI and creatinine were collected 24-48 hours before discharge. HR was measured after rest with patients in a supine position for 5–10 minutes or longer at 24 hours before discharge.

### Acoustic cardiography

EMATc was measured 24 hours before discharge. After rest in a supine position for 5–10 minutes, patients underwent acoustic cardiography (AUDICOR, Inovise Medical, Inc., Portland, OR, USA). EMATc was calculated by a computerized algorithm using simultaneous ECG and heart sound data obtained from the V3/V4 standard precordial position. Three independent readings were obtained for each patient, and average values were used in this study.

### Echocardiography

EF and LV end-diastolic diameter (LVEDD) were collected 24 hours before discharge and measured with the modified biplane Simpson’s rule (PHILIPS Affiniti 50).^8^ The researcher who interpreted the echocardiographic observations was blinded to all acoustic cardiographic observations and clinical data.

### Follow-up and endpoints

Of the 145 enrolled patients, 23 had cardiac death in hospital, one refused follow up, and one was lost to follow-up. All 120 included patients were followed up periodically by telephone call or out-patient visit every 2-3 months after discharge, and clinic visits for new symptoms were documented. Overall, the patients were followed up for 616.5 days (range: 202.75–875.25 days). The endpoints included cardiogenic death, all-cause death, and HF readmission. The definition of cardiogenic death included mortality caused by acute myocardial infarction or acute decompensated CHF that developed into cardiogenic shock or ventricular tachycardia/fibrillation. Sudden death occurring out of hospital was also included in cardiogenic death. Mortality for any reason was included in all-cause death. The definition of HF readmission included symptoms such as dyspnea, shortness of breath, ascites and chest pain that aggravated even after administration of oral diuretics, and acute coronary syndrome needing hospitalization. Patients were assigned to different groups based on the different endpoints.

### Statistical analysis

All statistical analyses were performed using SPSS, version 22.0 (SPSS, Inc., Chicago, IL, USA). Normally distributed continuous data are presented as mean±standard deviation (SD). Data with a non-normal distribution are presented as median with interquartile range [M (Q1, Q3)]. Continuous variables were compared with Mann–Whitney U test or two-sample Student’s t-test. Categorical variables are presented as proportion (frequency) and were compared with the Chi-square test. Independent risk factors for MACEs were identified by Cox regression analysis. EF and BNP were dichotomized with the upper normal limit, and Kaplan–Meier method was used to draw survival curves. P-values <0.05 were regarded as statistically significant.

Sample size calculation: according to methods reported in the literature,^7^,^9^ the EMATc of decompensated CHF is 15%. The all-cause death and admission rates of patients with stable CHF in 12 months were 17% and 44%, respectively. According to MERIT HF (the Metoprolol CR/XL Randomized Intervention Trial in congestive heart failure) post hoc analysis, the cardiac death rate with CHF NYHA II was 64%. We used a significance level α of 0.05, power (1-β) of 0.8, and two-sided test for power calculation. We planned to enroll 106 outpatients in the final study. Considering the possibility that 10% patients would drop out, we finally enrolled 120 patients.

## RESULTS

A total of 120 patients with an average age of 58.47±15.80 years were enrolled in this study. Among these patients, 14 patients had cardiogenic death, and 11 died from a non-cardiac cause. Hence, the total number of all-cause deaths was 25. Twenty-three patients were readmitted for acute onset of HF. Comparison of demographic data between different groups according to the endpoint is shown in Table-I.

**Table-I:** Comparison of clinic data between different groups according to endpoint.

	Cardiogenic death group (n=14)	Non-cardiogenic death group (n=106)	P	All-cause death group (n=25)	Surviving group (n=95)	P	HF readmission group (n=23)	Non-readmission group (n=72)	P
HR (bpm)	89 (79,101)	83 (72,100)	0.289	88 (74,100)	82 (72,100)	0.421	84 (72,100)	82 (72,100)	0.907
EF (%)	35.86±7.99	35.81 ± 8.53	0.511	37.56±6.75	35.59±8.47	0.285	32.83±8.18	36.47±8.43	0.073
LVEDD (mm)	64,14 ± 14.04	59.57 ± 8.25	0.098	59.08±11.85	58.40±9.24	0.758	59.81±8.30	57.94±9.53	0.401
EMATc (%)	15.09% ± 3.42	13.01 ± 3.44	0.098	13.21±3.32	13.25±3.61	0.958	13.80±3.65	13.08±3.60	0.408
EMATc>15%	8 (51.7%)	29 (27.4%)	0.032	8 (32.0%)	29 (30.5%)	0.887	7 (30.4%)	22 (30.6%)	0.991
Cr (µmol/L)	86.95 (63.93,101.75)	84.95 (71.22,106.53)	0.659	92.60 (71.25,111.45)	84.80 (70.40,104.80)	0.574	88.20 (70.40,106.00)	84.80 (70.33,104.78)	0.808
TnI (ng/ml)	0.050 (0.028,0.275)	0.110 (0.010,1.720)	0.436	0.270 (0.040,1.180)	0.070 (0.010,1.710)	0.200	0.040 (0.010,0.640)	0.095 (0.013,1.903)	0.160
BNP (pg/L)	703.5 (565.0,1087.0)	672 (165.25,1177.75)	0.205	1052.0 (635.5,1918.5)	552.0 (151.0,1014.0)	0.000	858.0 (316.00,1422.00)	371.50 (136.75,973.50)	0.023

HR-heart rate; EF- ejection fraction; LVEDD-left ventricular end-dilated diameter; EMATc-corrected electrical mechanical activated time ratio; Cr- Creatine; BNP-B type natriuretic peptide.

No significant differences in demographic and clinical parameters were observed between the cardiogenic death and non-cardiogenic death groups. The all-cause death group had a significantly higher mean age and a higher proportion with a past revascularization history than the surviving group. The readmission group had a significantly higher average age (66.61±11.95 years vs. 53.58±15.49 years, P≤0.001), proportion of patients with chronic renal failure, and proportion of patients treated with diuretics than the non-readmission group.

### Clinical data between different groups according to the endpoint.Table-I

The cardiogenic death group had a lower proportion of patients with an EMATc >15% than the non-cardiogenic death group [8(51.7%) vs. 29(27.4%), P=0.032]. The all-cause death group had a significantly higher BNP level [1052.0 (635.5,1918.5) vs. 552.0 (151.0,1014.0), P<0.001]. The HF readmission group had a higher serum BNP level [858.0 (316.00, 1422.00) vs. 371.50 (136.75, 973.50), P=0.023] than the non-readmission group.

Determination of risk factors for MACEs

**Fig.1:**
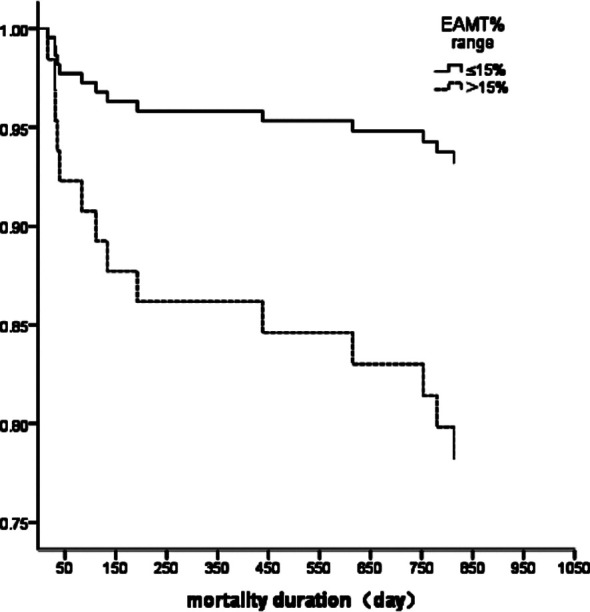
Survival curve for EMATc>15% in relation to cardiogenic death in CHF patients.

We performed Cox regression analysis with EF<40%, BNP>400 ng/L and EMATc>15% as variables to analyze the risk factors for cardiac death, all-cause death, and readmission due to HF. For cardiogenic death, only EMATc>15% was an independent risk factor (Table-II). The survival curve for EMATc>15% for cardiac death showed that the Log Rank χ2 value was 5.450 (P=0.02; Fig.1). For all-cause death, only BNP>400 ng/L was an independent risk factor for all-cause death in CHF patients (Table-II). For HF readmission, only BNP>400 ng/L was an independent risk factor for readmission due to HF (Table-II).

**Table-II:** Cox regression analysis of risk factors for MACEs.

Endpoint	Factor	Hazard ratio	95% CI	P
Cardogenic death	EMATc >15%	3.493	1.021–11.947	0.046
	EF <40%	0.500	0.110–2.269	0.369
	BNP >400 ng/L	2.210	0.673–7.260	0.191
All-cause death	EMATc >15%	1.021	0.414–2.515	0.964
	EF <40%	0.739	0.273–2.001	0.552
	BNP >400 ng/L	3.810	1.409–10.300	0.008
Readmission for HF	EMATc >15%	0.807	0.320–2.038	0.650
	EF <40%	1.158	0.367–3.654	0.802
	BNP >400 ng/L	2.746	1.095–6.885	0.031

## DISCUSSION

In this study, we found no significant difference in the mean EMATc between the cardiogenic death and non-cardiogenic death groups; however, an EMATc>15% was an independent risk factor for cardiac death in CHF patients, as revealed by Cox regression analysis and survival analysis.

EMAT is part of the pre-systolic phase and represents the time period needed for LV contraction to generate enough pressure to close the mitral valve.^10^ The EMATc is significantly prolonged in patients with decreased EF. Also, an EMATc >15% has a specificity of 94% and a sensitivity of 44% for the diagnosis of LV dysfunction, and was used as a criterion for left ventricular dysfunction.^6^,^11^ Our former study showed that elevated EMATc is an independent risk factor for in-hospital cardiogenic death, cardiogenic shock, and HF progression.^12^

It is well established that if the level of natriuretic peptide increases by 25%, the risk of MACEs and cardiac death increases substantially.^13^-^15^ Consistent with the above observations, we found that the BNP level was significantly higher in the all-cause death group and the HF readmission group than in the respective control groups. There was no significant difference in the BNP level between the cardiogenic death and non-cardiogenic death groups. Thus, it is highly recommended that EMATc>15% be used as a supplementary tool to evaluate the risk of cardiogenic death in CHF patients.

**Supplemental Table-I:** Comparison of demographic and baseline clinical data between different groups according to the dpoint.

	Cardiogenic death group (n=14)	Non-cardiogenic death group (n=106)	P	All-cause death group (n=25)	Surviving group (n=95)	P	HF readmission group (n=23)	Non-readmission group (n=72)	P
Sex, male	9 (9/14)	80 (75.5%)	0.351	16 (64.0%)	73 (76.8%)	0.206	16 (69.6%)	57 (79.2%)	0.398
Age (years)	60.57 ± 16.93	56.51 ± 14.79	0.585	65.04±14.71	56.74±8.47	0.019	66.61±11.95	53.58±15.49	0.000
Past myocardial infarction	5 (5/14)	22 (20.8%)	0.303	7 (28.0%)	20 (21.1%)	0.435	6 (26.1%)	14 (19.4%)	0.560
Past revascularization	4 (4/14)	11 (10.4%)	0.075	7 (28.0%)	8 (8.4%)	0.015	2 (8.7%)	6 (8.3%)	0.957
Hypertension	8 (8/14)	58 (54.7%)	0.864	15 (60.0%)	51 (53.7%)	0.655	13 (56.5%)	38 (52.8%)	0.813
Dilated myopathy	2(2/14)	6 (5.7%)	0.235	4 (16.0%)	4 (4.2%)	0.058	1(4.3%)	3(4.2%)	0.970
Diabetes	4 (4/14)	31 (39.2%)	0.958	7 (28.0%)	28(29.5%)	0.885	9 (39.1%)	19(26.4%)	0.296
Chronic renal failure	0 (0/14)	19 (17.9%)	0.123	3(12.0%_	16 (16.8%)	0.761	9(39.1%)	7 (9.7%)	0.003
β blocker	8 (8/14)	67 (63.2%)	0.771	14 (56.0%)	61 (62.4%)	0.491	14 (60.9%)	47 (65.3%)	0.804
ACEI/ARB	8 (8/14)	56 (52.8%)	0.785	11 (44%)	53 (55.8%)	0.369	12 (52.2%)	41 (56.9%)	0.810
Diuretics	12 (12/14)	78 (73.6%)	0.513	21 (84.0%)	69 (72.6%)	0.306	23 (100.0%)	46 (63.9%)	0.000
Weight (kg)	70 (52,77)	71 (65,81)	0.327	68.0 (57.0,74.0)	72.0 (65.0,81.5)	0.069	70.0 (61.3,80.5)	73.0 (65.5,83.5)	0.234

ACEI-angiotensin-converting enzyme inhibitor; ARB-angiotensin receptor blocker.

BNP levels are affected by many factors including old age and renal insufficiency.^16^-^18^ The ages of patients in the HF readmission group and all-cause death group were higher. CHF patients younger than 50 years have a <1% mortality rate, while those older than 80 years have an approximately 30% mortality rate.^19^ Consistent with the above findings, in the present study, the patients in the all-cause death group were older than those in the survival group, and the patients with HF readmission were older than the patients without readmission.

Resting echocardiography is still the most commonly used cardiac imaging method for CHF patients.^4^ Elevation of the EF is the most important imaging parameter that predicts better outcomes in CHF patients.^20^ In the present study, there was no significant difference in EF between the cardiogenic death and non-cardiogenic death groups. This discrepancy was likely due to a difference in patient selection. It is possible that there was bias related to the population selection. The average EF of our patients was already lower than that of patients in a previous study.^7^

### Limitations of this study

First, this study had a limited sample size, and thus, further studies are needed to corroborate our findings. Second, the follow-up duration was short. Third, some follow-ups were made by phone, making it difficult to monitor EMATc, BNP or EF for further comparison. Fourth, the enrolled patients included only those with an EF <50%; this study did not include patients with HF preserved ejection fraction (HFpEF). Fifth, the study was conducted between January 01, 2015 and January 01, 2018, during which time the angiotensin receptor-neprilysin inhibitor (ARNI) was not widely used in China. Therefore, we did not analyze the influence of the ARNI on the outcomes of CHF patients in this study.

## CONCLUSION

In conclusion, an EMATc>15% is an independent risk factor for cardiac death in stable CHF patients, can be used to predict the prognosis of stable CHF patients.

### Clinical Trial Registration:

http://www.chictr.org.cn/edit.aspx?pid=36214&htm=4

### Unique identifier:

ChiCTR1900021470.
